# Defects in Ultrasonic Vocalization of Cadherin-6 Knockout Mice

**DOI:** 10.1371/journal.pone.0049233

**Published:** 2012-11-16

**Authors:** Ryoko Nakagawa, Eiji Matsunaga, Kazuo Okanoya

**Affiliations:** 1 Laboratory for Biolinguistics, RIKEN Brain Science Institute, Wako, Japan; 2 Laboratory of Integrative Bioscience, Graduate School of Biomedical Sciences Hiroshima University, Hiroshima, Japan; 3 Laboratory for Symbolic Cognitive Development, RIKEN Brain Science Institute, Wako, Japan; 4 ERATO Okanoya Emotional Information Project, JST-ERATO, Wako, Japan; 5 Department of Life Sciences, Graduate School of Arts and Sciences, The University of Tokyo, Tokyo, Japan; 6 Emotional Information Joint Research Laboratory, RIKEN Brain Science Institute, Wako, Japan; Utrecht University, The Netherlands

## Abstract

**Background:**

Although some molecules have been identified as responsible for human language disorders, there is still little information about what molecular mechanisms establish the faculty of human language. Since mice, like songbirds, produce complex ultrasonic vocalizations for intraspecific communication in several social contexts, they can be good mammalian models for studying the molecular basis of human language. Having found that cadherins are involved in the vocal development of the Bengalese finch, a songbird, we expected cadherins to also be involved in mouse vocalizations.

**Methodology/Principal Findings:**

To examine whether similar molecular mechanisms underlie the vocalizations of songbirds and mammals, we categorized behavioral deficits including vocalization in cadherin-6 knockout mice. Comparing the ultrasonic vocalizations of cadherin-6 knockout mice with those of wild-type controls, we found that the peak frequency and variations of syllables were differed between the mutant and wild–type mice in both pup-isolation and adult-courtship contexts. Vocalizations during male-male aggression behavior, in contrast, did not differ between mutant and wild–type mice. Open-field tests revealed differences in locomotors activity in both heterozygote and homozygote animals and no difference in anxiety behavior.

**Conclusions/Significance:**

Our results suggest that cadherin-6 plays essential roles in locomotor activity and ultrasonic vocalization. These findings also support the idea that different species share some of the molecular mechanisms underlying vocal behavior.

## Introduction

The ability to speak and understand language is one of the most intellectual faculties of human beings. Although only humans are able to use language, components of language are seen in some nonhuman animals [1], [2]. Many studies investigating the neural basis of human language have therefore focused on the vocal communication of animals [3]–[12]. Songbirds have been used as animal models in studies investigating the brain mechanisms of complex vocalization including human language because they sing complex songs with sequential roles as human speech. The vocal processes and neural systems of songbirds have therefore been extensively analyzed at physiological, anatomical, and molecular levels. Although many analogies between humans and songbirds have been proposed with regard to vocal learning and the neural systems controlling those learning processes [4], [6], [13], [14], we need to also study mammalian model species if we are to attain a comprehensive understanding of the emergence of human language.

Mice produce ultrasonic successive vocalizations in social contexts as pup’s isolation calls and courtship calls [15]–[17], and the house mouse (*Mus musculus)* makes complex and lengthy vocalizations that [18], like birdsong and human speech, are based on sequential rules. It therefore seems that some basic neural foundation for the faculty of human language is conserved in mice. This makes them useful animal models for investigators studying language and searching for the molecular mechanisms of human language. Recently, several genes, which involved in language impairments or neurodevelopmental disorders including communication deficits, like autism spectrum disorders, were focused on as the mouse animal models [19]–[23]. Especially FoxP2 is focused on both songbird and mice field [19]–[22], and combining these studies it has been proposed that FoxP2 is associated with producing vocalizations in many animals, from songbirds to humans [24].

In our previous study using a songbird, the Bengalese finch, we found (1) that cadherin-6B (the avian ortholog of the mammalian cadherin-6) and -7 are expressed in vocal control areas and the expression of cadherin-7 in the robust nucleus of the arcopallium (RA) is downregulated during the sensorimotor learning stage [25] and (2) that lentiviral perturbation of cadherin expression in the RA produces severe defects in song development [26]. Cadherin is a cell adhesion molecule involved in synapse formation and function [27], and some cadherin-deficient mice show electrophysiological and behavioral defects [28], [29]. Suspecting that cadherins are involved not only in birdsong but also the ultrasonic vocalizations of mice, we analyzed the vocal and locomotor activity of cadherin-6 knockout (Cad6KO) mice.

**Figure 1:**
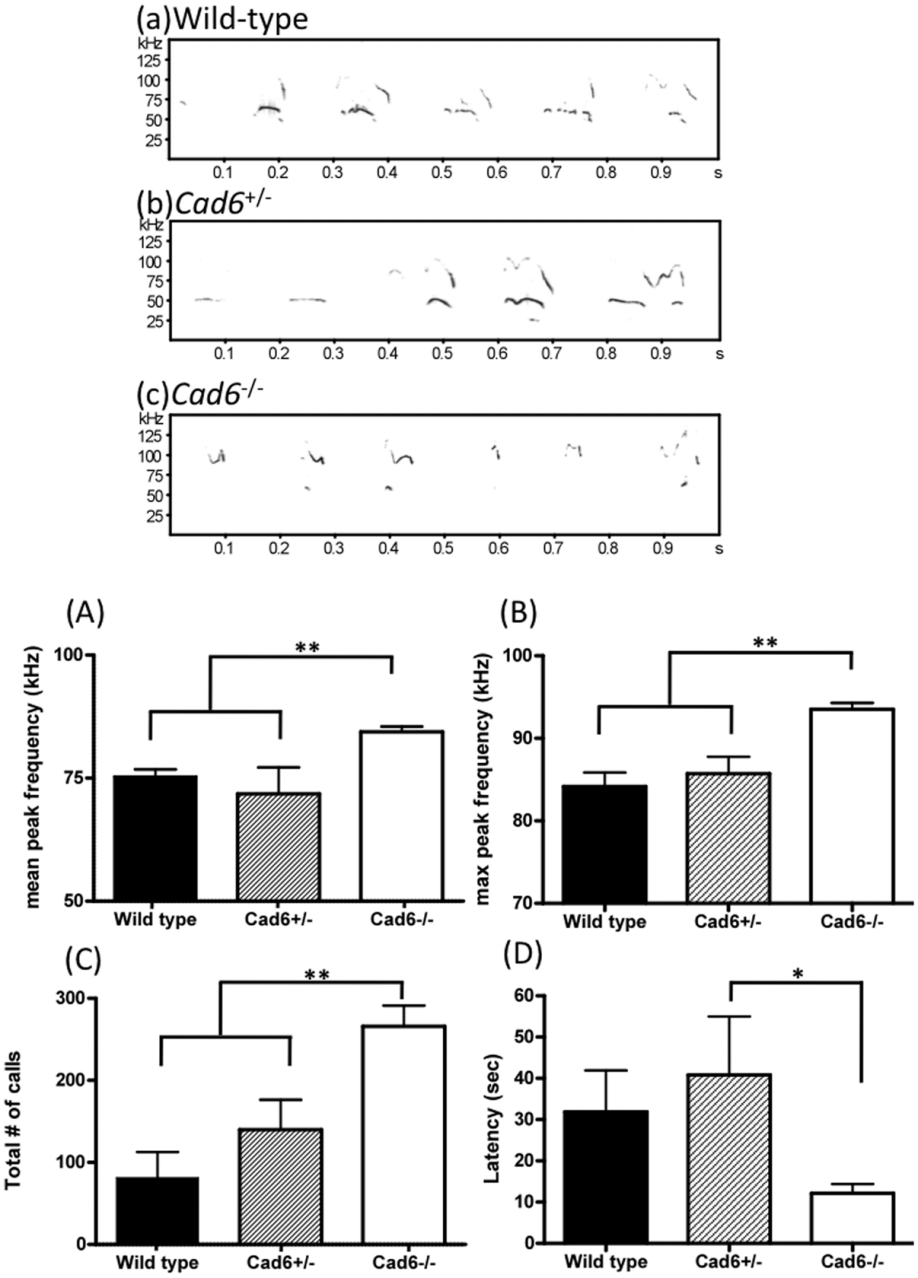
Ultrasonic vocalizations during pup isolation test. (a–c) Spectrograms (frequency, kHz * time, s) of isolation calls produced by each genotype. (A,B) Mean and maximum (max) peak frequency in the Cad6−/− group were much higher (>75 kHz) than in the Cad6+/− and wild-type groups (<75 kHz). (C) The number of calls by Cad6−/− pups was larger than that by either the Cad6+/− or wild-type pups. (D) The latency to start calling in the Cad6−/− group was shorter than that in the Cad6+/− group, and there was no significant difference between the Cad6−/− and wild-type groups. Error bars represent the SEM.

**Figure 2:**
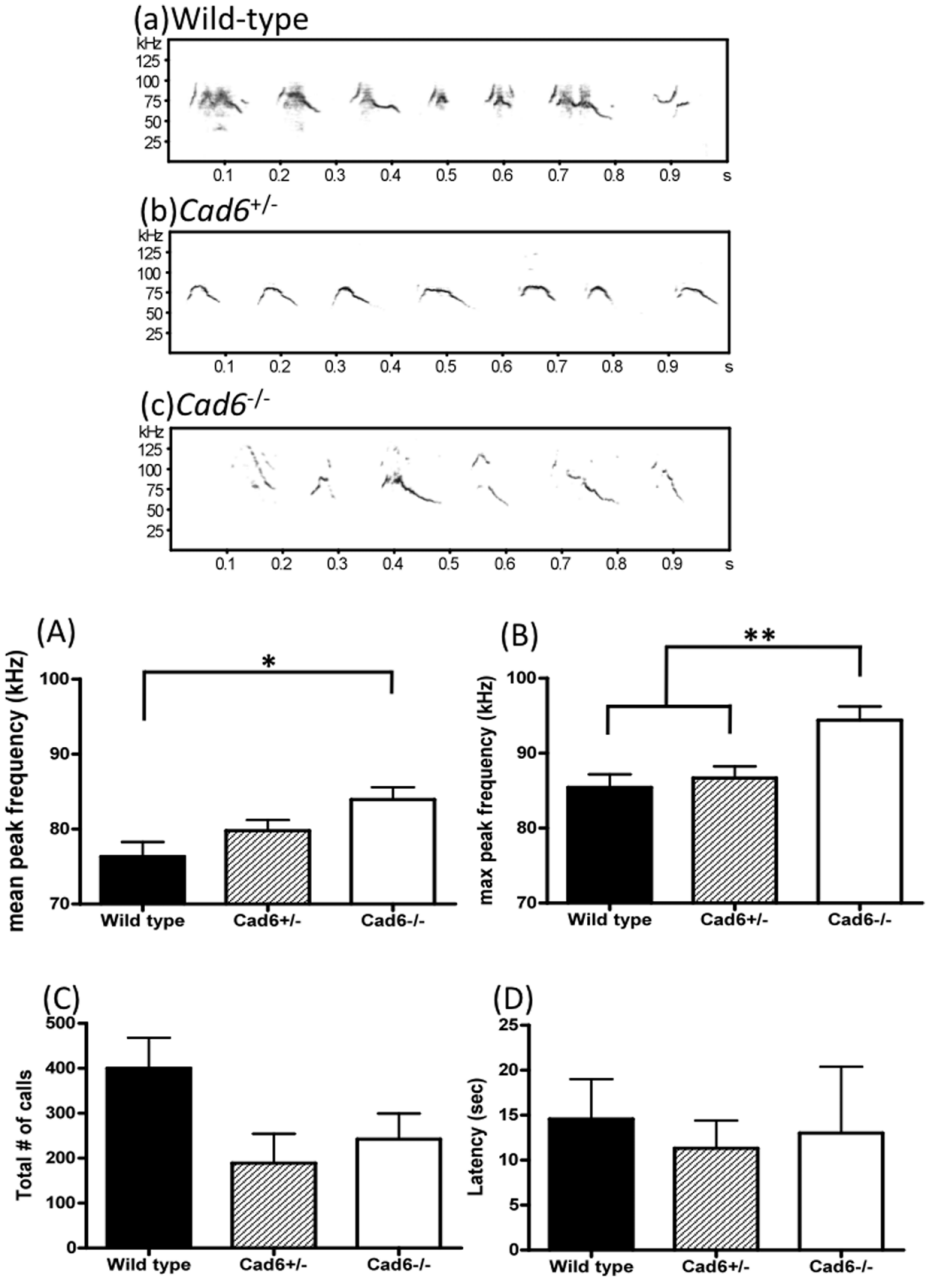
Ultrasonic vocalizations during courtship test. (a–c) Spectrograms (frequency, kHz * time, s) of courtship songs produced by each genotype. (A) Mean peak frequency during 3 min was higher for the in Cad6−/− group (>75 kHz) than the wild-type group (<75 kHz). (B) Maximum (max) peak frequency was higher for the Cad6−/− group than either the Cad6+/− or wild-type group. (C) The number of calls during 3 min did not differ significantly in the 3 groups. (D) The latency to start calling did not differ significantly in the 3 groups. Error bars represent the SEM.

## Results

### Basic Sound Features in Ultrasonic Vocalizations

Basic sound features, mean and max peak frequency, # of calls, latency to start calling in both pup’s isolation calls (Figure 1) and male’s courtship calls (Figure 2) were analyzed. Compared with WT and Cad6+/− mice, the mean and maximum peak frequencies of the syllables in Cad6−/− mice were significantly higher than those produced by WT mice [pup’s isolation call: Mean peak frequency: *F* (2, 48) = 13.01, *p*<.01; WT vs. Cad6+/−, *p*<0.01; WT vs. Cad6−/−, *p*<0.01. Maximum peak frequency: *F* (2, 48) = 15.27, *p*<.001; Cad6−/− vs. Cad6+/−, *p*<.001; Cad6−/− vs. WT, *p*<.01 (Figure 1A and B); male’s courtship call: Mean peak frequency: *F* (2, 23) = 5.00, *p*<.05, Cad6−/− vs. Cad6+/−, *p*<.05; Cad6−/− vs. WT, *p*<.05. Maximum peak frequency: *F* (2, 23) = 8.04, *p*<.01; Cad6−/− vs. Cad6+/−, *p*<.01; Cad6−/− vs. WT, *p*<.01 (Figure 2A and B)]. As for pup’s isolation call, the number of calls was larger than that by either of the other groups [*F* (2, 48) = 9.91, *p*<.01; Cad6−/− vs. Cad6+/−, *p*<.001; Cad6−/− vs. WT, *p*<.01] (Figure 1C), and the latency of the first calling by Cad6−/− pups was shorter than that of the first calling by WT pups [*F* (2, 48) = 3.91, *p*<.05; Cad6−/− vs. WT *p*<.05] (Figure 1D). As for the adult male’s courtship call, the number of calls and the latency of calling initiation, however, did not differ significantly between Cad6−/− and WT mice [*F* (2, 23) = 2.51, *n.s.*; *F* (2, 23) = 0.64, *n.s.*] (Figure 2C and 2D).

**Figure 3:**
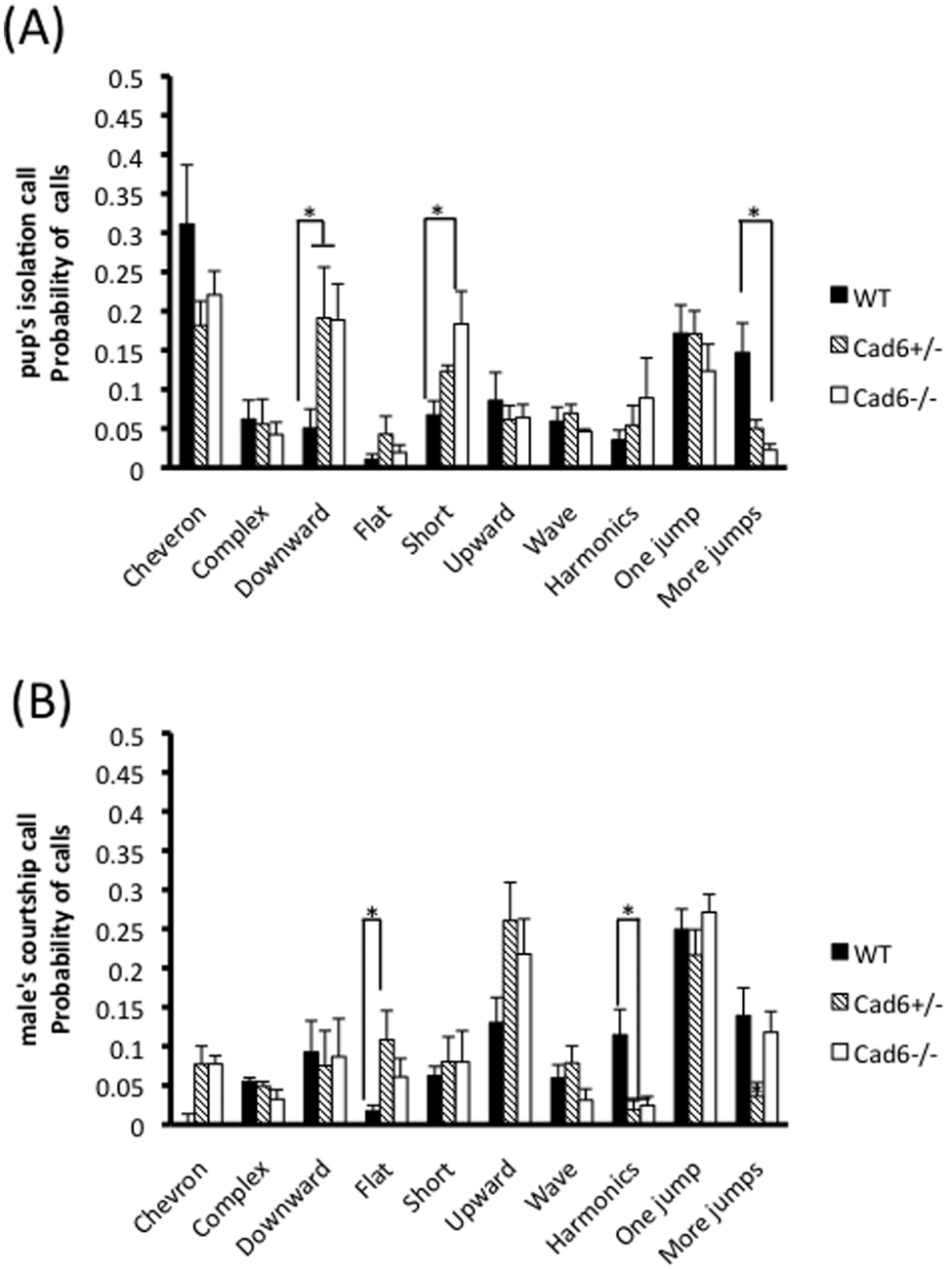
Repertoire of syllable categories in ultrasonic vocalizations. Probability of ultrasonic calls in each of the 10 different call categories in (A) pup’s isolation calls and in (B) male’s courtship calls. Cad6−/− pups emitted more “Downward” calls and “short” calls, and fewer “More jumps” calls than WT pups (A). Cad6−/− males produced few “Harmonics” calls (p<.05) than WT, and there are also differences between Cad6+/− and WT males in “Flat” calls (p<.05) and “More jumps” calls (p<.01). Error bars represent the SEM.

**Figure 4:**
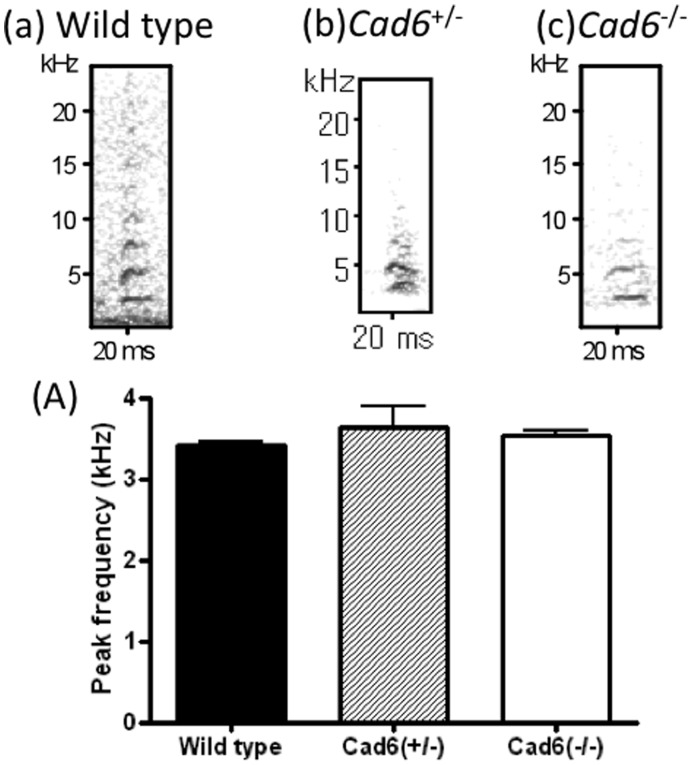
Audible vocalizations in male-male aggression behavior. (a–c) Spectrograms (frequency, kHz * time, s) of audible vocalizations produced by each genotype. (A) The peak frequency of audible vocalizations did not differ significantly between genotypes. Error bars represent the SEM.

**Figure 5:**
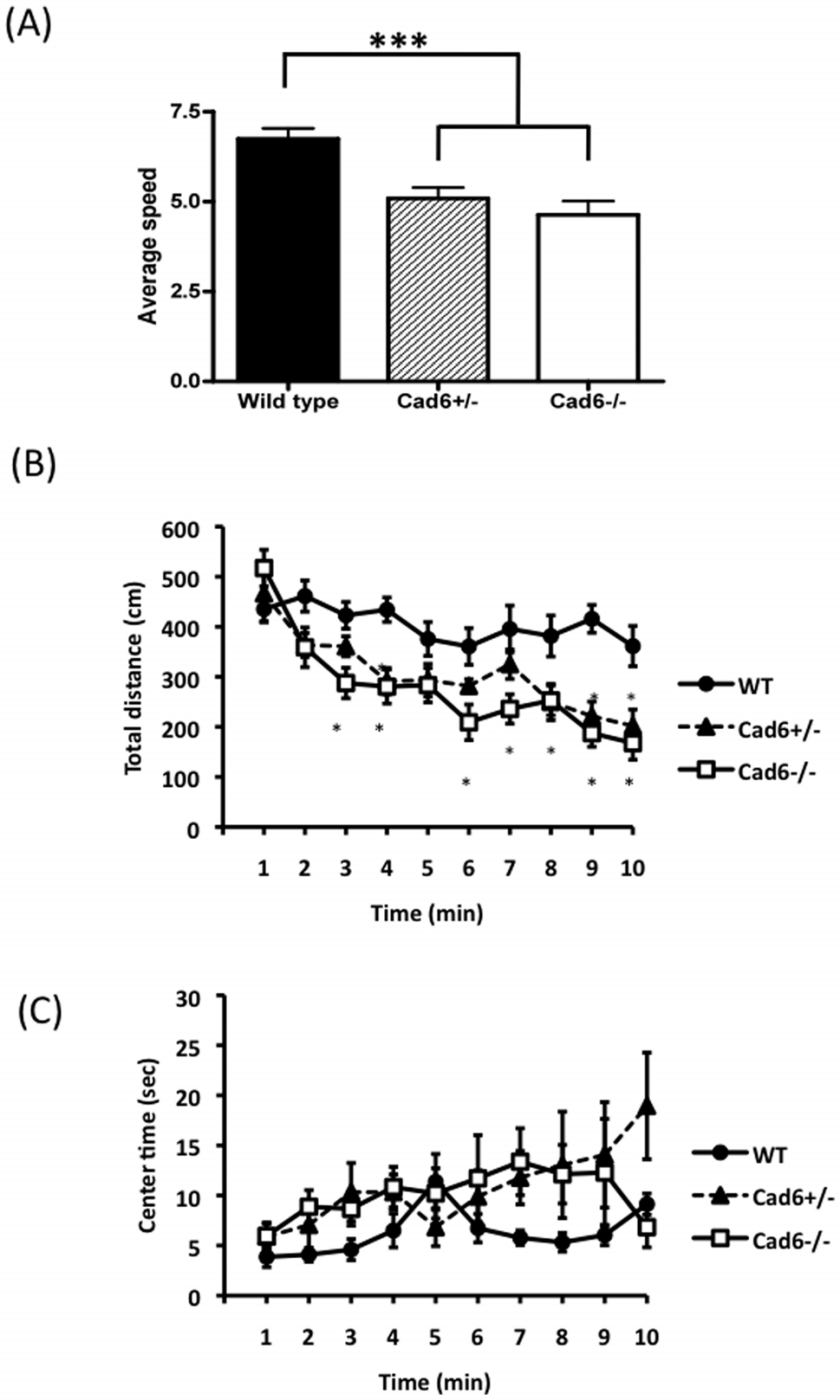
Locomotor activity in the open field test. (A) Running speed and (B) total distance were less than wild-type controls, but (C) the time spent in center did not differ significantly between genotypes. Error bars represent the SEM. (B),(C) Each data point represents the mean value in a one minutes bin.

### Comparison of Repertoire by Syllable Category

To investigate the quantitative deficits in Cad6KO mice, we categorized each syllable as 1 of 10 distinct categories: “Cheveron”, “Complex”, “Downward”, “Flat”, “Short”, “Upward”, “Wave”, “Harmonics”, “One jump”, “More jumps”. Syllable category differences in each genotype are shown in Figure 3.

In the pup’s isolation call, the pattern of call category differed across call category (*F*(9, 108) = 12.23, *p*<.01) and genotype × call category (*F*(18, 108) = 2.12, *p*<.01). Cad6−/− pups emitted more “Downward” calls (*p*<.01) and “short” calls (*p*<.05), and fewer “More jumps” calls (*p*<.01) than WT pups (Figure 3). In the male’s courtship calls, the pattern of call category also differed across call category (*F*(9, 108) = 12.34, *p*<.01) and genotype × call category (*F*(18, 108) = 2.17, *p*<.01). Cad6−/− males produced few “Harmonics” calls (*p*<.05) than WT, and there are also differences between Cad6+/− and WT males in “Flat” calls (*p*<.05) and “More jumps” calls (*p*<.01).

These results thus suggest that Cad6 knockout mice have defects extending the frequency range and control the vocal repertoire of both the ultrasonic courtship song and the ultrasonic isolation call.

### Male-male Aggression Call Test

Exploring the possibility of defects in vocalizations produced by Cad6KO mice in the aggression call test, we found that the peak frequency of vocalizations in aggression behavior is not differed between groups [*F* (2, 16) = 0.11, *n.s.*] (Figure 4A).

### Open-field Test

To investigate the possibility that the deficits of the vocalization related to the abnormality of locomotor activity or anxiety levels, we used the open-field test to examine the animals’ amounts of locomotor activity and their presumptive anxiety levels. Cad6−/− mice run more slowly than did Cad6+/− and WT mice [*F* (2, 29) = 12.14, *p*<0.01; Cad6−/− vs. Cad6+/−, *p*<0.01; Cad6−/− vs. WT, *p*<0.01] (Figure 5A). In addition, Cad6−/− mice exhibited less distance traveled [*F*(2,29) = 10.66, *p*<.01] (Figure 5B) The times spent in the corner and center areas during the 10-min open-field test, however, did not differ significantly between groups [*F* (2, 29) = 2.67, *n.s.*] (Figure 5C).

## Discussion

### Vocalization Defects of Ultrasonic Range and Moter Deficits in Cad6KO Mice

Analysis of the vocal behavior of Cad6KO mice revealed that both juvenile and adult homozygous mutant mice produced vocalizations with a higher pitch and unusual repertoire than did heterozygous and wild-type mice in both pup’s isolation calls and adult male’s courtship calls, but that vocalizations in male-male aggression behavior did not differ in these three groups. These results suggest that, as for vocalization behaviors, Cad6KO mice have defects only in the ultrasonic successive vocalizations, and that the defects are not caused due to impairment of peripheral vocal organs because they could vocalize in different social context. In addition, Cad6KO adult male mice showed deficits in the acoustic features and repertoire of calls but not the latency of vocalizations. This suggest that mechanisms controlling acoustic structures may be independent of the mechanisms controlling their motivation like how often and in which context do mice vocalize.

Since Cad6 is expressed in many brain areas of postnatal mouse brain–including the somatosensory cortex, motor cortex, and limbic system ([29], [30]; E.M. et al., unpublished data)–it is possible that Cad6KO mice have some general motor, somatosensory, or emotional defects. Indeed, pups showed anxiety response in the # of calls and latency to start calling. We therefore used the open-field test to examine the general motor activity and emotional state of the mutant mice. Both Cad6 homozygote and heterozygote mice demonstrated a decreased locomotors activity, however, the time spent in the center and corner areas in open field test suggested no anxiety differences between groups. It is possible that pup’s anxiety-like response in isolation calls is due to abnormal peak frequency of their USVs. Pup’s USVs are important for the development of mother-pup relationship [15]. Inhibits of dam’s aggression behaviors for pups by pup’s USVs [31] suggested that dam’s maternal care will be changed by pup’s abnormal vocalizations. Therefore, pup’s anxiety level may be related by their dam’s behavioral responses. In addition, locomotors activity deficits were observed not only in Cad6 homozygote but also in heterozygote mice. The motor deficits could be also associated with a controlling vocalization features, however, the deficits in peak frequency range are observed only in Cad6 homozygote mice. These results further suggest there is still the possibility that cadherin6 play some roles in mouse vocalization.

### Possible Molecular Basis of the Faculty of Human Language and Involvement of Cadherin Superfamily

Many genes responsible for human language impairment–such as Robo1, KIAA0391, DCD2, and Dyx1C1–have been identified by linkage analysis of human patients [32]–[34]. These genes control neuronal migration and axon guidance. In addition, MRI diffusion tensor imaging shows that the brains of people with innate alexia exhibit neural network defects such as reduced nerve fibers in the lateral hemisphere [35]. Thus, genes regulating cell migration or specific neural circuit formation may play essential roles in the neural basis of human language. As we describe above, cadherin-6 expressed in many brain areas such as the somatosensory cortex, motor cortex, limbic system, and it seems also in ambiguous nucleus ([30]; E.M. et al., unpublished data). Previous study reported that singing-related immediate early genes expressed in mice cingulated, motor cortex and the anterodorsal striatum [36]. Combing the result of previous study and our study, it is possible that cadherin expression in motor cortex is related to defects in mouse USVs. To identify the singing-related brain areas and neural circuits, further studies will be necessary using such as the electroporation technique or viral vectors to knockdown the gene expression in a region specific manner.

In this study, we found by analyzing Cad6KO mice that Cad6 is essential for proper ultrasonic vocalization. Many studies have shown that type-II cadherins (e.g., Cad6) are localized in the synapse and involved in synapse formation and function [28], [29], [37]–[41], so cadherins are assumed to control vocalizations by regulating synapse formation and function not only in mice but also in humans.

Recently several researches proposed possibilities that mice ultrasonic vocalizations are basically innate [42], [43] though mice have slight vocal learning ability to modify the pitch [36], [44]. Although brain mechanisms for vocalization differ between vocal learners and non-vocal learners [4], [45]–[49], previous FoxP2 studies and our present study suggest that some molecular constraints might have existed during the convergent evolution of vocal systems in birds, mice and humans. Combining mouse studies with songbird studies we will enable us to fully understand the molecular mechanisms of human language, so genetically modified mammalian animals should be powerful tools helping us understand the whole spectrum of molecular mechanisms of human language.

## Materials and Methods

### Animals

Cad6KO mice [50] were kindly provided by Dr. Masatoshi Takeichi. They are derived from C57BL/6 JJcl mice and bred with WT mice purchased from Japan Clea Co. Ltd. (Yokohama, Japan) in our laboratory. Food and water were given ad libitum, and all animals were kept at constant temperature (23±2°C) and humidity (55%±10%) under a 12-h light/dark cycle (light on at 08:00). Animals were genotyped by polymerase chain reaction (PCR) using the following primers: Cad6-Neo (5′-CCTGCTTGCCGAATATCATGGTGGAAAATG-3′), Cad6-11 (5′-ACCGGTACTTCTTGCTGCTGCTCTTTTGGGTCG-3′), and Cad6-228r (5′-GTAACTTGCCCACGTACTGATAATCGGATC-3′). The PCR condition was 38 cycles of 94° for 1 min, 65° for 2 min, and 72° for 1 min 30 s. All experiments were approved by RIKEN’s Animal Care and Use Committee and conformed to National Institutes of Health Guidelines.

### Behavioral Analysis

The vocal behavior of Cad6KO mice was assessed by examining both ultrasonic and audible vocalizations, and their locomotor activity was assessed by open-field testing.

Vocalizations were examined under three conditions: (1) pup isolation (2) male courtship, and (3) male-male aggression context.

#### (1) Pup-isolation test

Fifty-one mice [26 Cad6−/−, 14 Cad6+/−, 11 wild type (WT)] were used on postnatal day 7. After each pup was removed from its huddling littermates and put into a 500-mL plastic beaker placed in a soundproof box, its vocalizations were recorded for 3 min. To maintain the pup’s body temperature, absorbent cotton was placed in the beaker. Condenser microphones (CM16/CMPA, Avisoft Bioacoustics, Berlin, Germany) 10 cm above the animal were connected, through a pre-amplifier (Avisoft Ultrasound Gate 416H; Avisoft Bioacoustics, Berlin, Germany), to a personal computer. Signals were recorded to the hard disk via Avisoft-Recorder USGH (Avisoft Bioacoustics, Berlin, Germany) set at a 300-kHz sampling rate, and the recorded sound was stored as “.wav” files.

#### (2) Male-courtship test

Twenty-six mice (8 Cad6−/−, 8 Cad6+/−, and 10 WT) 13–17 weeks old were tested. Five WT female mice were used as stimulus animals. The stimulus mice were surgically ovariectomized seven days before the test, and estrogen was administrated chronically via a silastic tube. In each test trial the experimental male was placed in a plastic cage 30 s before a randomly selected stimulus female was put into the cage and vocalizations were recorded for 3 min using the same equipment used in the pup-isolation test.

#### (3) Male-Male aggression test

Non-successive vocalizations in the lower pitch as the human audible range were also examined in a male-male aggression behavior test. 24 weeks old 19 animals (5 Cad6−/−, 7Cad6+/−, and 7 WT) are used as experimental subjects, and 5 WT mice used as intruders. Five weeks before the test the experimental animals and stimulus animals (i.e., intruders) were isolated in the breeding cages. Three days before the test the pharyngeal nerves of the stimulus animals were surgically extirpated. The audible vocalization test was performed in a plastic cage with a condenser microphone located 30 cm above and centered over the floor of the soundproof box. In each trial the experimental animal was put into the test cage 30 s before the stimulus animal was and recording then proceeded for 5 min.

#### Open-field test

The open-field test is commonly used to determine general activity levels, gross locomotor activity, and exploration habits in mice. We used it to examine whether the Cad6 knockout animals show abnormal behavior as measured by the amount of activity and emotional behavior. Thirty-two 8-week-old mice (12 Cad6−/−, 9 Cad6+/−, and 11 WT) were tested. Each animal was placed in the center of the open-field box (50 cm×50 cm×40 cm high) and allowed to move freely for 10 min while being tracked by a system using ImageJ software. The floor of the box was separated into center and corner areas by virtual lines making a 5*5 grid, and in each 10-min trial the total distance traveled, mean running speed, and time spent in the center (10 cm from the wall) were recorded. The floor of the open-field box was cleaned with 70% ethanol after every trial.

### Sound Analysis

The recorded files were transferred to SASLab Pro (ver. 4.52, Avisoft Bioacoustics, Berlin, Germany) for fast Fourier transform analysis (FFT length 512, 100% frame size, 100% frame size, Hamming window, 50% time window overlap) with a 20-kHz high-pass filter. In both the isolation and courtship contexts we analyzed the number of syllables, the latency to start calling, and the mean peak frequency of each syllable. In the audible vocalization test we analyzed only mean peak frequency after background noise was reduced by the GoldWave program.

Waveform pattern of syllables were analyzed in the sonograms collected from every genotype (pup’s isolation call: 531 WT syllables, 1188 Cad6+/− syllables; 1724 Cad6−/− syllables; adult male’s courtship call: 1931 WT syllables, 408 Cad6+/− syllables, 1777 Cad6−/− syllables). Each call is categorized as the 1 of 10 distinct categories, based on internal pitch change, length, and shape, according to previously reported categories with minor modifications [51].

### Statistical Analysis

One-way or two-way analysis of variance (ANOVA) with Tukey’s honestly significant difference (HSD) test was used for statistical analysis. Probability of vocalizations was standardized by angular transformation before analyzed.
